# Identification of Distinct Molecular Patterns and a Four-Gene Signature in Colon Cancer Based on Invasion-Related Genes

**DOI:** 10.3389/fgene.2021.685371

**Published:** 2021-08-06

**Authors:** Yunfei Dong, Tao Shang, HaiXin Ji, Xiukou Zhou, Zhi Chen

**Affiliations:** Department of Proctology, The First Affiliated Hospital of Zhejiang Chinese Medical University (Zhejiang Provincial Hospital of Traditional Chinese Medicine), Hangzhou, China

**Keywords:** colon cancer, invasion, gene signature, subtypes, The Cancer Genome Atlas, prognostic

## Abstract

**Background:**

The pathological stage of colon cancer cannot accurately predict recurrence, and to date, no gene expression characteristics have been demonstrated to be reliable for prognostic stratification in clinical practice, perhaps because colon cancer is a heterogeneous disease. The purpose was to establish a comprehensive molecular classification and prognostic marker for colon cancer based on invasion-related expression profiling.

**Methods:**

From the Gene Expression Omnibus (GEO) database, we collected two microarray datasets of colon cancer samples, and another dataset was obtained from The Cancer Genome Atlas (TCGA). Differentially expressed genes (DEGs) further underwent univariate analysis, least absolute shrinkage, selection operator (LASSO) regression analysis, and multivariate Cox survival analysis to screen prognosis-associated feature genes, which were further verified with test datasets.

**Results:**

Two molecular subtypes (C1 and C2) were identified based on invasion-related genes in the colon cancer samples in TCGA training dataset, and C2 had a good prognosis. Moreover, C1 was more sensitive to immunotherapy. A total of 1,514 invasion-related genes, specifically 124 downregulated genes and 1,390 upregulated genes in C1 and C2, were identified as DEGs. A four-gene prognostic signature was identified and validated, and colon cancer patients were stratified into a high-risk group and a low-risk group. Multivariate regression analyses and a nomogram indicated that the four-gene signature developed in this study was an independent predictive factor and had a relatively good predictive capability when adjusting for other clinical factors.

**Conclusion:**

This research provided novel insights into the mechanisms underlying invasion and offered a novel biomarker of a poor prognosis in colon cancer patients.

## Introduction

Colon cancer, which is a malignant tumor of the digestive tract derived from the mucosal epithelium of the colon or rectum, has become the third most frequent cancer among men and the second most frequent cancer among women worldwide (Arnold et al., 2017; Althobaiti and Jradi, 2019). Colon cancer starts insidiously, progresses rapidly, and has a poor prognosis and high mortality rate. There are many treatments available to prolong the survival of patients with advanced disease, and surgery is the main treatment for colon cancer, but the 5-year survival rate is 50% (Zhai et al., 2017). In total, 15–20% of colon cancer patients relapse after treatment (Shi et al., 2014), and CRC recurrence after therapeutic surgery is a major obstacle in improving the overall survival rate of colon cancer patients (Gerger et al., 2011). As a highly heterogeneous disease, colon cancer involves DNA repair defects, DNA methylation, chromosome instability, and other molecular pathogeneses in the course of disease development. Biomarkers have been used as common tools for disease detection and prognosis management in colon cancer patients (Juo et al., 2014). Therefore, the determination of molecular changes in colon cancer patients has become a hotspot in colon cancer research.

With the development of the Human Genome Project and the arrival of the post-genome era, the development of various high-throughput biomedical technologies has led to the exponential growth of biological data, which are currently mostly applied to tumor functional genomics. Based on biological information service platforms for the construction of public networks and constantly emerging and accumulating biomedical information and clinical data resources, an increasing number of studies have focused on these vast amounts of genetic research and data processing, using bioinformatic analysis to mine the expression data for tumor-associated genes involved in the pathogenesis, progression, and changes in the process of transformation, to find CRC-related changes in the genome, obtain CRC gene expression profiles, and improve the CRC diagnosis threshold, which is of great value for the clinical application of genetic information (Tutar et al., 2015). Transcriptomic analysis has been widely used to describe the prognostic characteristics of colon cancer patients and has produced many candidate biomarkers with potential clinical value (Marisa et al., 2013; Xu et al., 2017; Wei et al., 2018). However, small sample sizes and certain technical factors restrict the consistency of the proposed signatures and provide limited prognostic information. In addition, the high heterogeneity of colon cancer makes it important to establish a reliable signal to identify patients with a high risk of disease recurrence. To this end, the integration of results from multiple studies is expected to yield more reliable prognostic characteristics.

We, therefore, attempted to determine and validate a robust prognosis-related feature by integrating multiple datasets from colon cancer patients. This research developed a four-gene signature with solid prognostic performance for colon cancer that may complement traditional clinical prognostic factors and provide effective therapeutic interventions and individualized therapies for treating colon cancer patients.

## Materials and Methods

### Sources of Obtained Data

Clinical follow-up information and RNA-Seq data (FPKM) for colon cancer (COAD) were downloaded from TCGA database^1^ (Author Anonymous, 2018). The expression spectrum was converted to TPM, genes with low expression (genes with less than 1 transcript in more than 50% of all samples) were removed, and Ensembl IDs were converted into gene symbols. The median value was taken as the expression spectrum of gene symbols when multiple Ensembl IDs corresponded to the same gene symbol. Log2 conversion was performed for the expression spectrum data. Two datasets, GSE17538 (Smith et al., 2010) and GSE38832 (Tripathi et al., 2014), in the MINiML format were acquired from the GEO database^2^, both of which were sequencing data generated on the GPL570 platform ([HG-U133_Plus_2] Affymetrix Human Genome U133 Plus 2.0 Array). The chip data set was converted from probes to gene symbols according to the GPL570 annotation file (the middle value was taken as the expression spectrum of the gene symbol when multiple probes corresponded to the same gene symbol; probe expression was removed when there were multiple gene symbols per probe). The microarray dataset included only colon cancer tumor samples with survival time and survival status. The clinical information available after data preprocessing is shown in Table 1. The invasion-related gene set was derived from the c2.all.v7.0.symbols.gmt file on the GSEA website^3^ (Subramanian et al., 2005). There were a total of 1,202 genes involved in the 11 pathways related to invasion.

**TABLE 1:** Clinical information of three datasets.

	TCGA	GSE17538	GSE38832
**Status**			
Alive	320	145	83
Dead	117	42	9
**T Stage**			
T1	11		
T2	75		
T3	300		
T4	50		
TX	1		
**N Stage**			
N0	256		
N1	103		
N2	78		
**M Stage**			
M0	323		
M1	60		
MX	54		
**Stage**			
I	73	28	18
II	167	70	35
III	126	75	39
IV	60	14	
X	11		
**Gender**			
Female	203	88	
Male	234	99	
**Grade**			
G1		16	
G2		134	
G3		21	
GX		16	
**Lymphatic_invasion**			
NO	242		
YES	152		
Unknown	43		
**Age**			
>65	255	98	
= 65	182	89	

### Molecular Typing Based on Invasion-Related Genes

In TCGA dataset, univariate Cox analysis of the 1,202 invasion-related genes was performed using the Coxph function of the R package survival (V3.1–12), and the genes associated with colon cancer prognosis were obtained (*p* < 0.01). Next, the R package NMF (V1.48.0) (Li and Ngom, 2013) was used to conduct molecular typing of colon cancer samples from TCGA data set, and the optimal typing was selected.

### Comparison of Clinical Features and Molecular Mutations Between Molecular Subtypes

The chi-square test was used to identify differences in clinical characteristics between the two molecular subtypes of colon cancer. According to the SNV/indel results of MuTect detection in TCGA database, the “maftools” (Mayakonda et al., 2018) software package was used for the mutation annotation format (MAF) on the basis of TCGA queue.

### Comparison of Molecular Subtypes With Existing Molecular Subtypes

A total of six categories of immune infiltration were identified in human tumors based on corresponding tumor-promoting and tumor suppressor factors, namely, C6 (TGF-beta dominates), C5 (immunologically silent), C4 (lymphocyte depletion), C3 (inflammation), C2 (INF-γ predominant), and C1 (wound healing).

### Analysis of Molecular Subtypes With Immune Scores and Immunotherapy Outcomes

First, the R software package MCPcounter (V1.2.0) (Dienstmann et al., 2019) was used to determine the immune cell scores of each sample, and then differences in the immune cell scores of molecular subtypes were compared. In recent years, research on immune checkpoint inhibition (ICI) has achieved breakthroughs in clinical response in a variety of human cancers, but most cancer patients do not benefit from ICI therapy. Studies have reported that a clinical response to an anti-PD-1 antibody, a type of ICI, is more likely to occur in tumors that already have T-cell infiltration and PD-L1 expression. Moreover, IFN-α functions critically in regulating the expression of PD-L1. High levels of IFN-, accompanied by accelerated lymphocyte infiltration, may be the key to recognition of tumor-cytotoxic immunophenotypes, which may lead to treatment with anti-PD-1 therapy. We compared the expression of PDCD1 (PD-L1) and IFNG (IFN-α) among molecular subtypes and calculated the Pearson correlation coefficient of PDCD1 and IFNG expression. We also calculated the Pearson correlation coefficients between the expression of these two genes and immune scores for T cells and CD8 T cells. The above analyses identify our molecular subtypes based on the potential related to immunotherapy.

### Analysis and Functional Identification of DEGs in Molecular Subtypes

In TCGA dataset, the R software package limma (V3.44.3) (Ritchie et al., 2015) was applied to analyze DEGs in the expression spectrum data of molecular subtypes, and an FDR < 0.01 and an |FC| > 1.5 were used as the threshold to screen and filter the differentially expressed genes. The R package clusterProfiler (V3.16.0) (Yu et al., 2012) was used to perform GO functional annotation and KEGG pathway enrichment analyses of differentially upregulated and downregulated genes, and an FDR < 0.05 was used as the threshold for filtering.

### Detection of Prognostic Genes and Their Characteristics

LASSO, univariate regression, and multivariate regression analyses were performed to examine the relationships between the expression of invasion-related genes and the overall survival (OS) of colon cancer patients. In the univariate Cox regression analysis, a gene with a *p*-value < 0.05 was considered a candidate prognostic gene. Multivariate analysis, LASSO penalization, and stepAIC were applied for subsequent screening. Each gene was evaluated to determine its regression coefficient and hazard ratios (HRs), and the qualified mRNAs were ultimately included in a signature for colon cancer.

### Establishment of a Prognostic Model Based on Invasion-Related Genes

Prognostic prediction by a signature for colon cancer patients was evaluated based on the expression of each optimal prognostic mRNA multiplied by the relative regression coefficient weight, which was calculated from the multivariate model with the following formula:

$$\text{RiskScore}=\sum_{i=1}^{n}co\text{e}f\left(i\right)\times gene\left(i\right)$$

where *coef*(*i*) refers to the coefficient of the *i*th gene, and *gene*(*i*) refers to the expression level of the *i*th gene. Each sample was evaluated to obtain a risk score value, and the risk score cutoff was set as the middle value. Samples with a risk score greater than the middle value were considered high-risk samples, and those with a risk score less than or equal to the middle value were considered low-risk samples. The Kaplan–Meier (KM) survival curves for the two groups were plotted. A receiver operating characteristic (ROC) curve for OS prediction was constructed to evaluate the specificity and sensitivity of the model. Cox multivariate analysis of clinicopathological characteristics of colon cancer patients was also conducted to examine prognostic model independence.

### Verification of the Prognostic Risk Model

Patients were classified into a high-risk or low-risk group after comparing the risk scores of TCGA training set, the entire TCGA cohort, and two independent external datasets, the GSE17538 and GSE38832 cohorts. The cutoff values were calculated from the training cohort. KM curve, multivariate Cox, and time-dependent ROC analyses were also performed. Additionally, stratified analyses were performed based on clinicopathological characteristics.

### Nomogram

A nomogram and calibration curve were established by the R language “RMS” software package (Jiang et al., 2019). The consistency between the predicted probability and actual observed frequency was assessed by determining correctness. Next, the performance of the nomogram was visualized by showing the predicted and observed results in the calibration curve, with the 45° line representing the most accurate prediction.

## Results

### Identification of Molecular Subtypes of Colon Cancer

**FIGURE 1:**
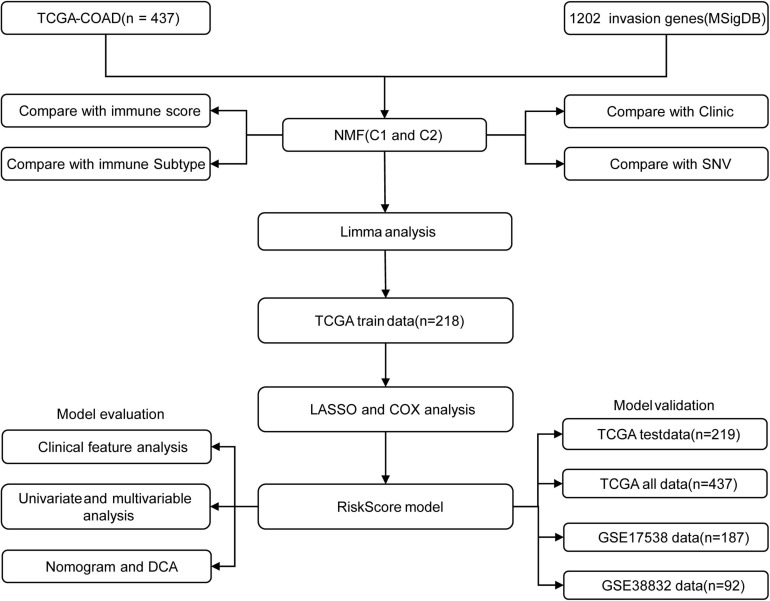
The study flow chart.

**FIGURE 2:**
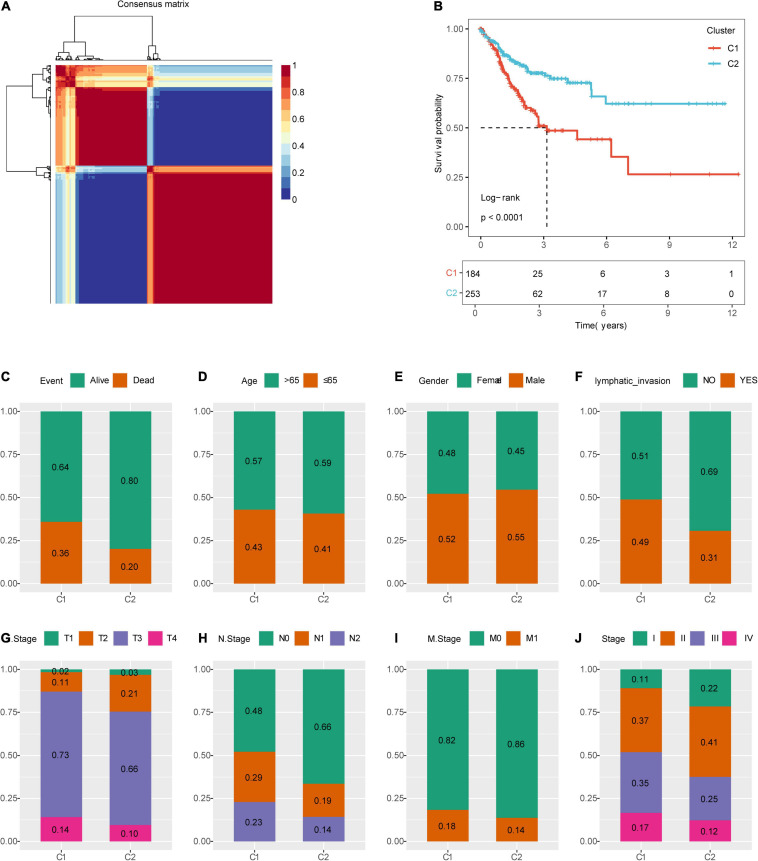
Identification of molecular subtypes of colon cancer. **(A)** NMF clustering (*K* = 2). **(B)** Kaplan–Meier (KM) curves of the molecular subtypes. **(C)** Comparison of the distribution of survival states among molecular subtypes. **(D)** Comparison of the distribution of aging states among molecular subtypes. **(E)** Comparison of the distribution of sex among molecular subtypes. **(F)** Comparison of the distribution of lymphatic invasion states among molecular subtypes. **(G)** Comparison of the distribution of T states among molecular subtypes. **(H)** Comparison of the distribution of N states among molecular subtypes. **(I)** Comparison of the distribution of M states among molecular subtypes. **(J)** Comparison of the distribution of stages among molecular subtypes.

Figure 1 shows the study flow chart. Univariate Cox survival analysis was performed on the expression profiles of 1,132 invasion-related genes using the Coxph function of the R package survival (V3.1–12), and 56 genes were identified to be associated with the prognosis of colon cancer (*p* < 0.01). Functional annotation analysis was carried out on 56 genes, which were related to exosomes, signal transduction, and transporters. The 56 prognostic genes were used to cluster TCGA samples with the R package NMF (*K* = 2–10), and TCGA samples were classified into a C1 or C2 category according to the clustering results (Figure 2A). KM survival curve analysis showed that the molecular subtype C2 had a better prognosis than C1 (Figure 2B). The distribution of clinical trait statuses between the two subtypes showed that dead samples, lymphatic invasion, and the incidences of T2, T3, and T4 in the prognostic C1 subtype were significantly higher than those in the prognostic C2 subtype, while N0 and stage I samples were significantly less common in the prognostic C1 subtype than in the prognostic C2 subtype (Figure 2C).

### Comparison of Mutations Between Molecular Subtypes and Existing Immune Subtypes

**FIGURE 3:**
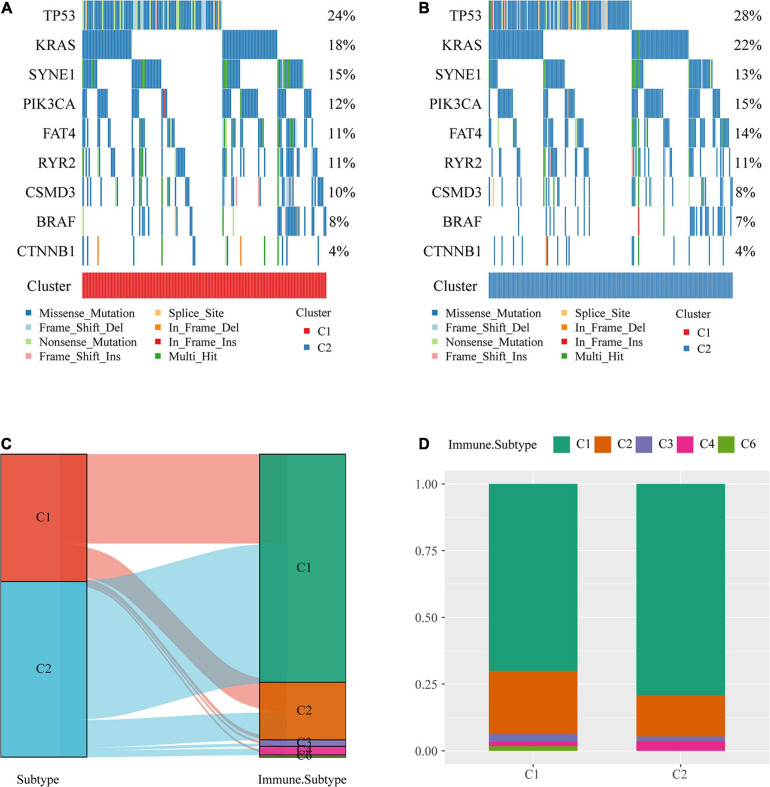
Comparison of mutations among molecular subtypes and existing immune subtypes. **(A)** Key gene mutation map of the molecular subtype C1. **(B)** Key gene mutation map of the molecular subtype C2. **(C)** Sankey diagrams of the study molecular subtypes and existing molecular subtypes. **(D)** Distribution of existing molecular subtypes within our molecular subtypes.

The profiles of key mutation genes in colon cancer, such as TP53, KRAS, SYNE1, PIK3CA, BRAF, FAT4, CSMD3, CTNNB1, and RYR2, were selected, and the mutation frequencies of the SYNE1, CSMD4, and BRAF genes in the C1 subtype were higher than those in the C2 subtype, whereas the mutation frequencies of TP53 (Mirgayazova et al., 2019), KRAS, PIK3CA, and FAT4 in the C1 subtype were lower than those in the C2 subtype (Figures 3A,B).

The majority of colon cancer patients recorded in TCGA dataset were in the C1 or C2 immune subtype (approximately 94.3%) compared with the existing immune subtypes, with the C1 immune subtype having a better prognosis than the C2 immune subtype, and the C5 immune subtype was absent from TCGA colon cancer dataset (Figure 3C). Additionally, we compared the distribution of these subtypes across our metabolic subtypes and found that the immune subtype C1 was predominant over our C2 subtype, which was consistent with a better prognosis for our C2 subtype (Figure 3D).

### Evaluation of Immune Cell Scores and Immunotherapy Outcomes

**FIGURE 4:**
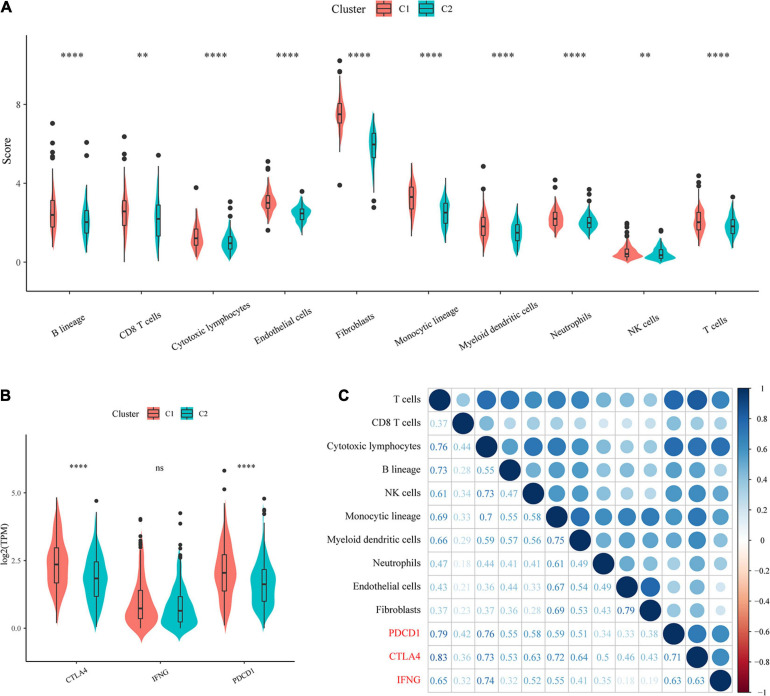
Evaluation of immune cell scores and immunotherapy outcomes. **(A)** Comparison of immune scores and immune checkpoint gene expression between molecular subtypes. **(B)** Comparison of immune checkpoint gene expression between molecular subtypes. **(C)** Correlations of immune cell scores with immune-related genes.

To identify the relationships of immune cell scores with the two molecular subtypes, first, the immune cell scores of each sample were calculated separately using the R software package MCPcounter, and then the differences in immune cell scores between the molecular subtypes were compared. The results showed that the 10 immune cell scores were higher for the C1 subtype than for the C2 subtype, which included scores for T cells and CD8 T cells (Figure 4A).

In recent years, immune checkpoint inhibition (ICI) research has led to breakthroughs in clinical response in a variety of human cancers, yet the majority of cancer patients do not benefit from ICI. Studies have demonstrated that the clinical response to anti-PD-1 antibodies, a type of ICI, is more likely to occur in tumors that already have T-cell infiltration and PD-L1 expression. Additionally, IFN-γ has an important role in regulating PD-L1 expression, and high levels of IFN-γ accompanied by accelerated lymphocyte infiltration may be critical for recognizing the immune phenotype of tumor cytotoxicity, which could potentially indicate anti-PD-1 therapeutic efficacy.

We compared the expression of the three genes PDCD1 (PD-L1), CTLA4, and IFNNG (IFN-γ) between the molecular subtypes and observed that compared with the C2 subtype, the C1 subtype showed significantly higher PDCD1 and CTLA4 expression (Figure 4B). In addition, we calculated the Pearson correlation coefficients between PDCD1, CTLA4, and IFNG gene expression and immune cell scores and found that they showed strong positive correlations (Figure 4C). The above results suggested that our molecular subtype C1 may respond better to immunotherapy than C2.

### Identification of Differentially Expressed Genes

**FIGURE 5:**
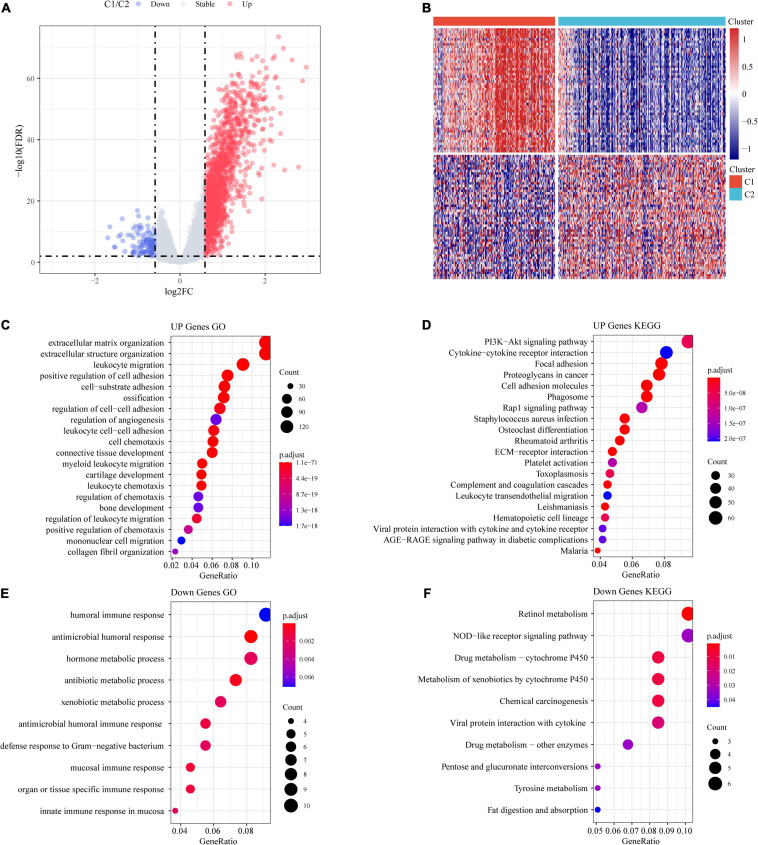
Identification of differentially expressed genes (DEGs). **(A)** Volcano maps of DEGs. **(B)** Heatmaps of DEGs. **(C)** GO functional analysis of differentially upregulated genes. **(D)** KEGG functional analysis of differentially upregulated genes. **(E)** GO functional analysis of differentially downregulated genes. **(F)** KEGG functional analysis of differentially downregulated genes.

A total of 1,514 DEGs from the C1 and C2 molecular subtypes were determined by the limma package (Figures 5A,B). Furthermore, the 1,390 upregulated differentially expressed genes and 124 downregulated differentially expressed genes related to the colon cancer subtype grouped by the R software package ClusterProfiler (v3.16.0) were used for GO functional enrichment analysis and KEGG pathway analysis. Here, the GO data demonstrated that the 1,390 upregulated genes were primarily involved in extracellular matrix organization, myeloid leukocyte migration, positive regulation of cell adhesion, and another 1,307 pathways (Figure 5C).

From the results of the KEGG pathway enrichment analysis, it was found that the 1,390 upregulated genes were related to proteoglycans, focal adhesion, the PI3K-Akt signaling pathway, cancer, and another 70 pathways (Figure 5D).

The GO results showed that the 124 downregulated genes were primarily involved in the innate immune response, antimicrobial humoral immune response, humoral immune response in the mucosa, and 19 other pathways (Figure 5E). The results of the KEGG pathway enrichment analysis demonstrated that the 124 downregulated genes were related to drug metabolism-cytochrome P450, the NOD-like receptor signaling pathway, retinol metabolism, and another seven pathways (Figure 5F).

### Establishment of a Prognostic Risk Scoring System With Four Genes

**FIGURE 6:**
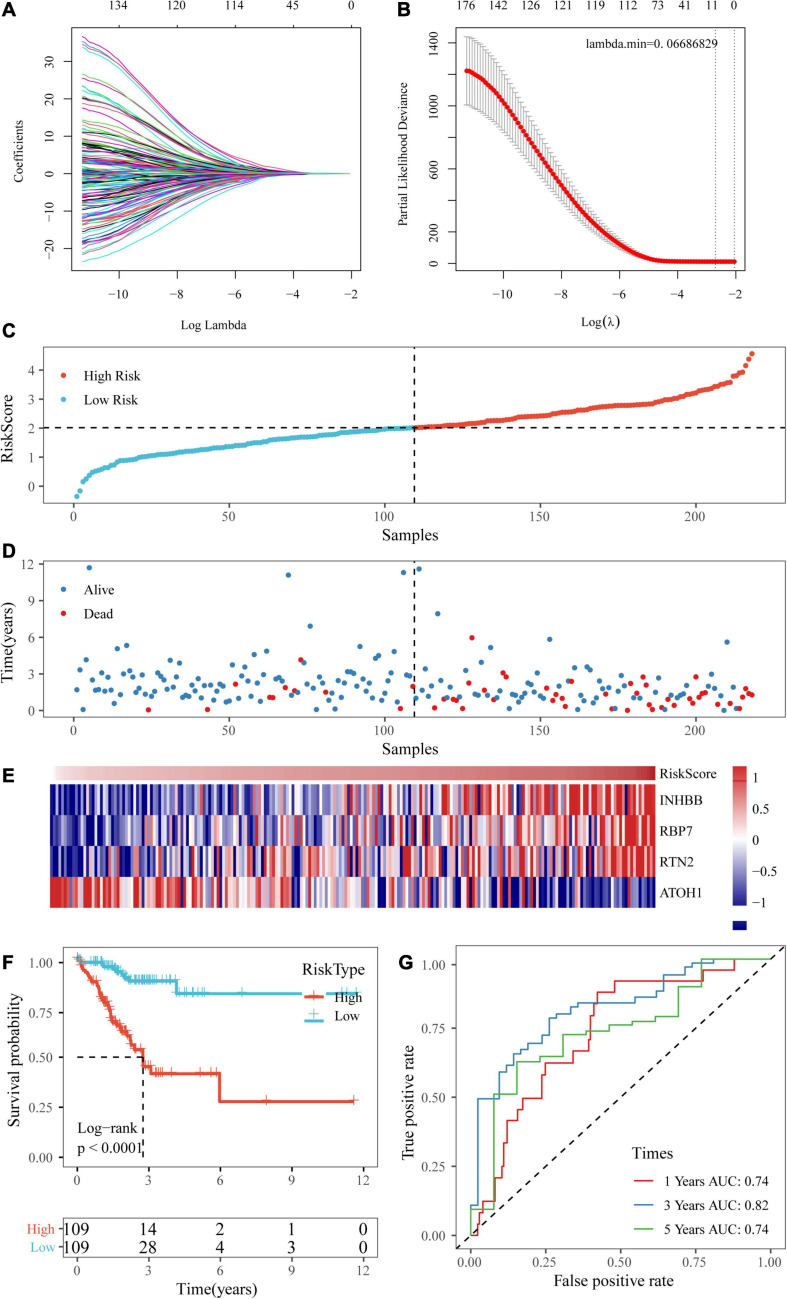
Establishment of a prognostic risk scoring system based on four genes. **(A,B)** A larger *λ* indicating a greater penalty for the linear model with more variables. **(C,D)** The distribution of risk scores in TCGA training dataset and the distribution of corresponding survival states. **(E)** Heatmaps of gene expression in the risk score model. **(F,G)** KM curves and ROC curves of the risk score in TCGA training datasets.

Univariate Cox regression analysis was performed on the 1,514 DEGs, and 139 genes associated with colorectal cancer prognosis were detected. Genes that might have been highly correlated with other genes were excluded by LASSO regression. The degree of complexity for LASSO regression was calculated by the parameter lambda (*λ*), with a larger *λ* indicating a greater penalty for the linear model with more variables (Figure 6A). When *λ* = 0.06686829 (Figure 6B), 10 candidate genes were acquired by LASSO regression. For the training cohort, multivariate Cox regression analysis showed that the independent prognostic factors were INHBB, RBP7, RTN2, and ATOH1 (Table 2), which were, therefore, used to build a prognostic model risk score, which had the formula (0.268 × INHBB expression value) + (0.225 × RBP7 expression value) + (0.514 × RTN2 expression value) + (−0.205 × ATOH1 expression value).

**TABLE 2:** Risk factors for the risk model.

Gene	Coef	HR	HR (lower, 0.95)	HR (upper, 0.95)	*P*
INHBB	0.268	1.308	1.090	1.569	0.004
RBP7	0.225	1.252	0.961	1.632	0.096
RTN2	0.514	1.672	1.203	2.325	0.002
ATOH1	−0.205	0.815	0.702	0.945	0.007

Next, the risk scores of the samples in TCGA training data set were obtained based on the calculation formula of the risk score. Then, the median was taken as the cutoff point. If the risk score was higher than the median, the sample was considered high risk; otherwise, the sample was considered low risk. The risk score of the four-gene signature and patient survival are shown in Figures 6C,D. The gene expression heatmap indicated that INHBB, RBP7, and RTN2 were risk factors and that ATOH1 was a protective factor (Figure 6E). Also, we used RT-qPCR and Western blot assay to validate the level of genes in model in two colorectal cancer cell line. The data showed that mRNA and protein expressions of INHBB, RBP7, and RTN2 were higher, while ATOH1 was downregulated in SW480 cells and HT29 cells in comparison with FHC cells (Supplementary Figures 1A,B). The log-rank test and KM survival curve analysis revealed that patients in the high-risk group tended to have a poor prognosis in TCGA training dataset (Figure 6F). The AUCs of the ROC curves for 1-year survival and 5-year survival were both 0.74, and the AUC for 3-year survival was 0.82 (Figure 6G).

### Verification of the Four-Gene Signature With Internal Datasets

To assess the robustness of our four-gene signature, we validated the signature in a test dataset and the entire TCGA dataset.

**FIGURE 7:**
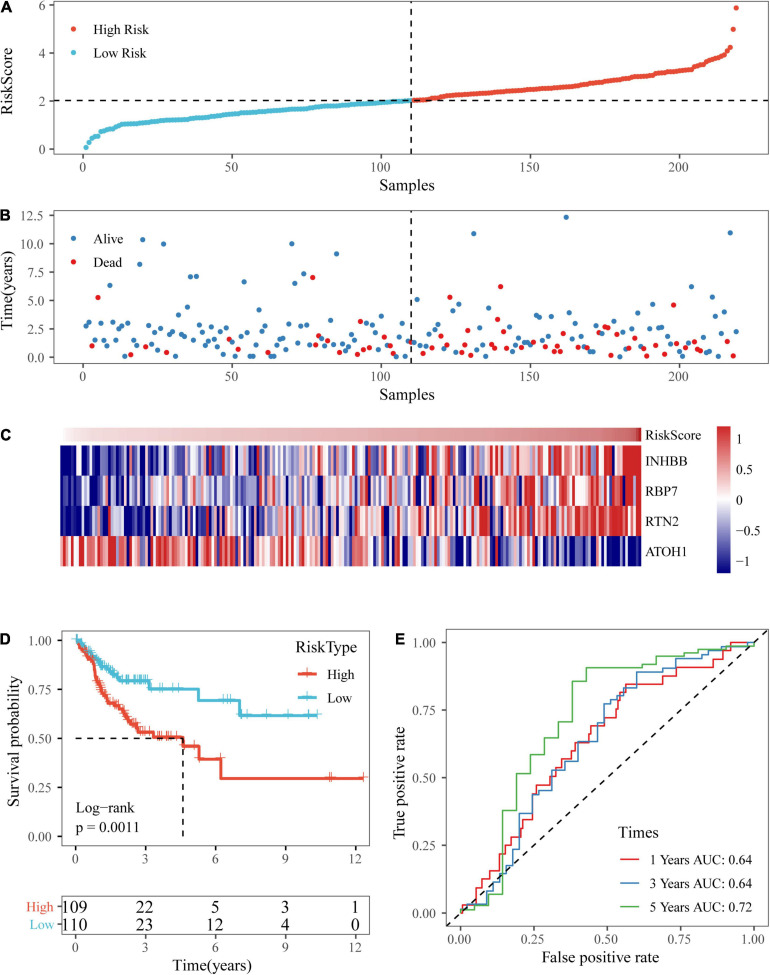
Verification of the four-gene signature in TCGA test datasets. **(A,B)** The distribution of risk scores in TCGA test dataset and the distribution of corresponding survival states. **(C)** Heatmaps of gene expression in the risk score model. **(D,E)** KM curves and ROC curves of the risk score in TCGA test datasets.

**FIGURE 8:**
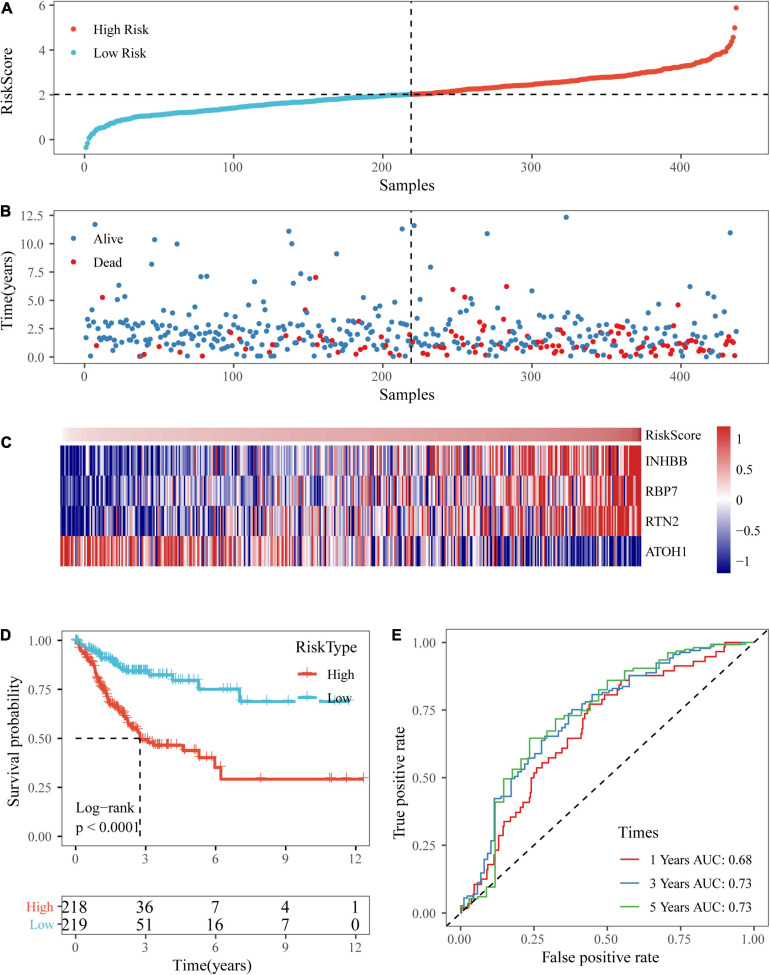
Verification of the four-gene signature in entire TCGA datasets. **(A,B)** The distribution of risk scores in the entire TCGA dataset and the distribution of corresponding survival states. **(C)** Heatmaps of gene expression in the risk score model. **(D,E)** KM curves and ROC curves of the risk score in entire TCGA datasets.

Based on the above formula, the survival risk scores of patients in the test set were determined. Figures 7A–C display patient survival, a gene expression heatmap for the test set, and the risk score of the four-gene signature. The KM curve demonstrated a significant difference in prognosis between the low-risk group and the high-risk group (*p*-value of the log-rank test = 0.0011; Figure 7D). From the time-dependent ROC curve data, the four-gene signature was shown to effectively predict the OS of colon cancer patients (Figure 7E). Moreover, a gene expression heatmap for the entire TCGA dataset, patient survival results, and the risk score of the four-gene signature are shown in Figures 8A–C. The KM curves showed a significant difference in survival time between the patients in the high-risk group and those in the low-risk group (log-rank test *p*-value < 0.0001, Figure 8D), and the AUCs were 0.68, 0.73, and 0.73 for 1, 3, and 5 years for the entire TCGA dataset (Figure 8E).

### Validation of the Four-Gene Signature in External Datasets

To further verify the accuracy of our risk model with different platforms and different data sets, our risk model was verified with the two independent data sets GSE17538 and GSE38832.

**FIGURE 9:**
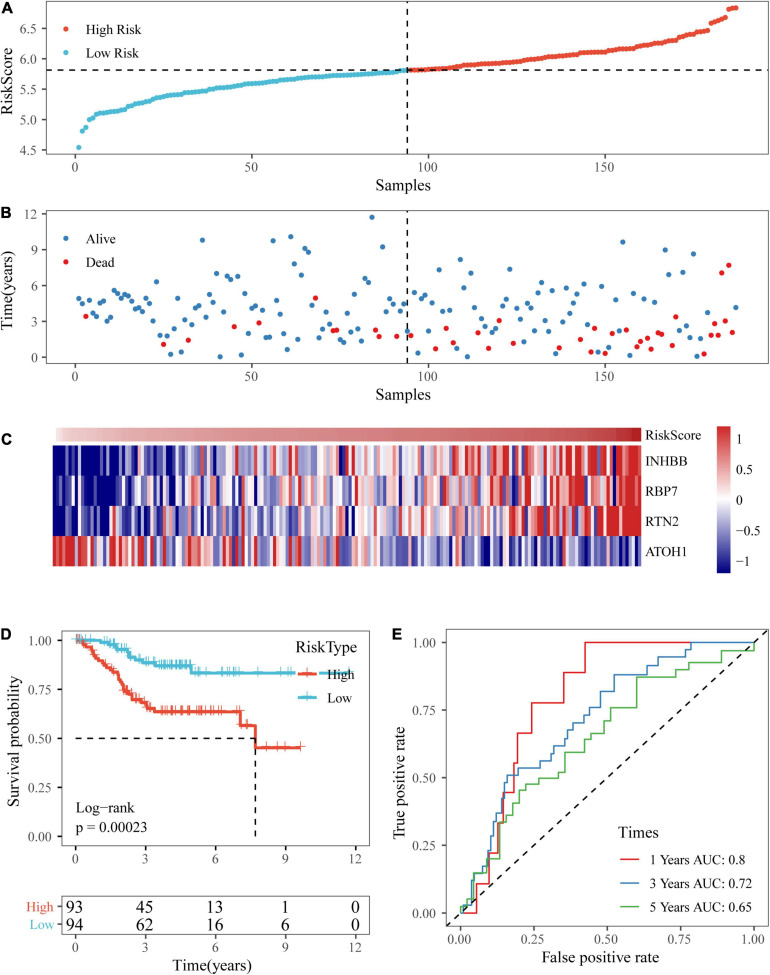
Verification of the four-gene signature in the GSE17538 dataset. **(A,B)** The distribution of risk scores in the GSE17538 dataset and the distribution of corresponding survival states. **(C)** Heatmaps of gene expression in the risk score model. **(D,E)** KM curves and ROC curves of the risk score in the GSE17538 dataset.

**FIGURE 10:**
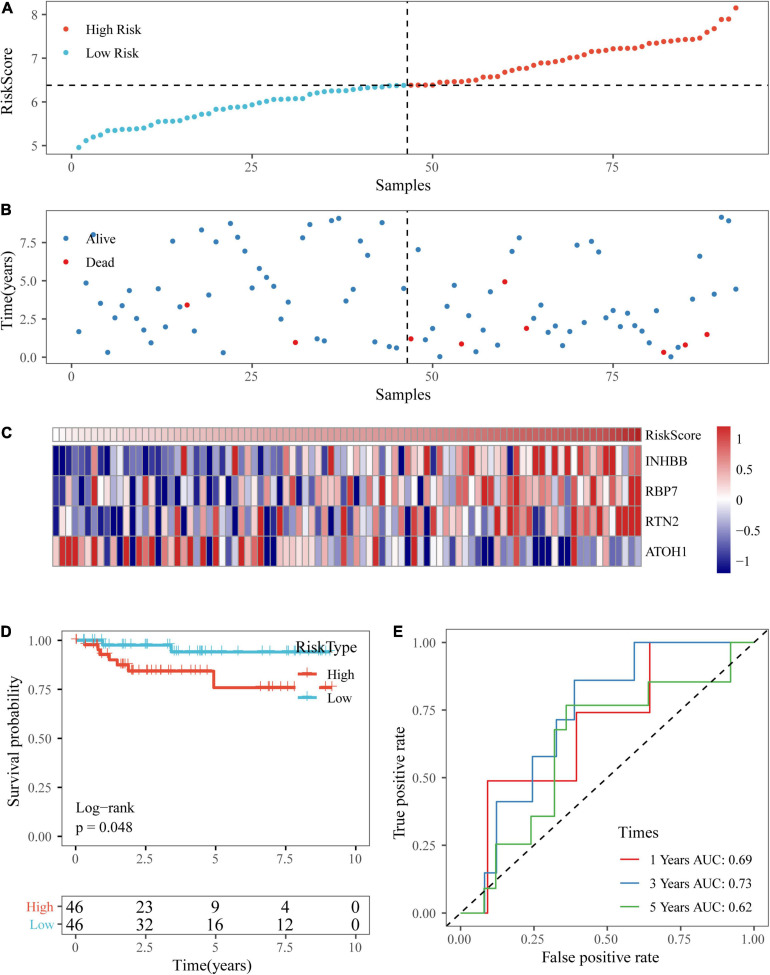
Verification of the four-gene signature in the GSE38832 dataset. **(A,B)** The distribution of risk scores in the GSE38832 dataset and the distribution of corresponding survival states. **(C)** Heatmaps of gene expression in the risk score model. **(D,E)** KM curves and ROC curves of the risk score in the GSE38832 dataset.

Based on the above formula, we calculated the survival risk scores of patients in the test set. Patient survival results, the risk score of the four-gene signature, and a gene expression heatmap for the GSE17538 dataset are displayed in Figures 9A–C. The KM curve showed a significant difference in the prognosis of patients between the high-risk group and the low-risk group (log-rank test *p*-value = 0.0011; Figure 9D). The time-dependent ROC curve results showed that this four-gene signature could effectively predict the OS of colon cancer patients (Figure 9E). Moreover, the patient survival results, risk score of the four-gene signature, and gene expression heatmap for the GSE38832 dataset are shown in Figures 10A–C. The KM curves indicated a significant difference in the survival time of patients between the high-risk and low-risk groups (Figure 10D), and the AUCs were 0.69, 0.73, and 0.62 for 1, 3, and 5 years in TCGA dataset (Figure 10E).

### Analysis of Clinical Characteristics of the Risk Model

**FIGURE 11:**
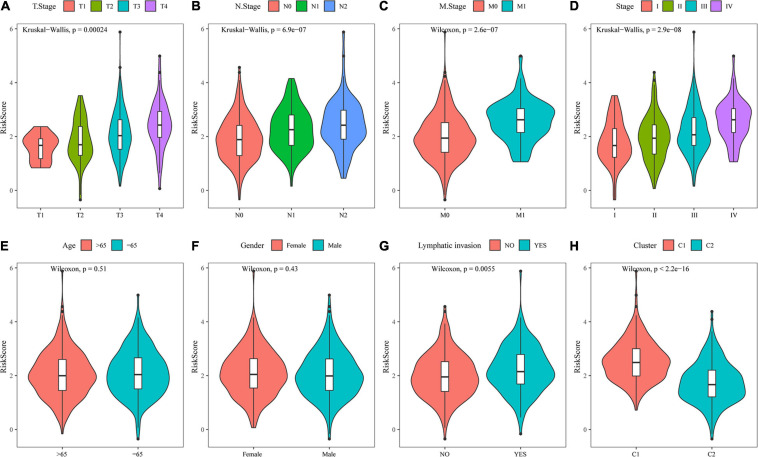
Analysis of clinical characteristics of the risk model. **(A)** Comparison of the risk score between T stage subsamples. **(B)** Comparison of the risk score between N stage subsamples. **(C)** Comparison of the risk score between M stage subsamples. **(D)** Comparison of the risk score between stage subsamples. **(E)** Comparison of the risk score between age subsamples. **(F)** Comparison of the risk score between sex subsamples. **(G)** Comparison of the risk score between lymphatic invasion subsamples. **(H)** Comparison of the risk score between samples grouped by molecular subtype.

By comparing the distribution of risk scores among clinical feature groups in the entire TCGA dataset, it was found that there were significant differences in the T stage, N stage, M stage, stage, lymphatic invasion, and our molecular subtypes (*p* < 0.05). No differences in age or sex grouping were detected. In the lymphatic invasion-grouped samples, the samples with invasion had a higher risk score. Between our molecular subtypes, the risk score of the C1 subtype, which had a worse prognosis, was significantly higher than that of the C2 subtype, which had a better prognosis (Figure 11). The model also showed good classification of chemotherapy- and radiotherapy-treated samples (Supplementary Figures 2A–C).

### The Risk Model Is an Independent Indicator of Colon Cancer Prognosis

Univariate and multivariate analyses were conducted to compare the prognostic prediction of risk parameters with clinicopathological parameters (Table 3). According to the present data, RiskType and M stage were determined to be two independent indicators for colon cancer prognosis, as they showed significant differences in the two analyses. We also determined the two analyses on the GSE17538 and GSE38832 datasets, and the results showed that RiskType was also an independent indicator in prediction of colon prognosis in two GEO datasets (Supplementary Tables 1 and 2).

**TABLE 3:** Univariate and multivariate analyses of entire TCGA dataset.

Feature	Univariable analysis				Multivariable analysis			
	HR	95% CI of HR		*P*	HR	95% CI of HR		*P*
		lower	upper			lower	upper	
Age	0.939	0.65	1.359	0.74	1.293	0.83	2.014	0.256
Gender	1.247	0.863	1.803	0.24	0.932	0.613	1.416	0.741
T Stage	4.037	1.88	8.672	<1e-5	1.67	0.702	3.973	0.246
N Stage	2.723	1.876	3.951	<1e-5	0.444	0.156	1.265	0.129
M Stage	6.009	3.988	9.055	<1e-5	2.921	1.691	5.045	<1e-5
lymphatic_invasion	2.378	1.612	3.509	<1e-5	1.502	0.934	2.414	0.093
Stage	3.21	2.176	4.736	<1e-5	2.911	0.924	9.174	0.068
RiskType	3.43	2.272	5.179	<1e-5	2.398	1.459	3.94	0.001

### Nomogram and Its Clinical Application

**FIGURE 12:**
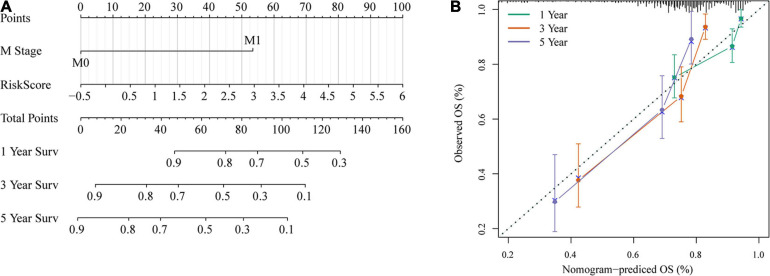
Nomogram and its clinical utility. **(A)** Nomogram. **(B)** Nomogram correction chart.

To provide a quantitative method for the prediction of OS in colon cancer patients, we constructed a nomogram based on the risk score and M stage, which were identified as independent prognostic factors by multivariate analyses (Figure 12A). Risk score features had the greatest impact on survival prediction. The calibration curves of 5-, 3-, and 1-year survival showed that the nomogram was almost an ideal model in terms of predicting colon cancer prognosis (Figure 12B). These results further supported the reliability of the prognostic model. Additionally, we screened four published signatures (Tian et al., 2017; Xu et al., 2017; Dai et al., 2018; Mo et al., 2019). Their risk scores were first calculated, and then the 1-, 3-, and 5-year AUCs of our model were compared with those of the four published models. The results showed that the 1-, 3-, and 5-year AUCs of our model were higher than those of the other four models (Supplementary Figures 2D–F). DCA also indicated that our model had better performance (Supplementary Figure 2G).

## Discussion

Differences in CRC tumors can affect the prediction of clinical treatment outcomes. The accuracy of patient clinical outcome prediction will be improved by classifying gene expression profiles and identifying subtypes of colorectal cancer with effective prognostic markers, which also provide valuable guidance for appropriate therapeutic interventions (Bramsen et al., 2017). CRC subtypes could advance accurate diagnosis and facilitate drug development. Many attempts have been made to use gene expression datasets to achieve this goal (Muzny et al., 2012; Ren et al., 2016). In a study by Bramsen et al. (2017), the use of a subtype strategy for CRC transcriptional profiling to identify molecular subtype-specific biomarkers helped to improve patient prognosis. Recent studies have established different subtype classifications based on the three molecular pathways that have been identified: chromosomal instability (CIN), CIMP, and microsatellite instability—high (MSI-H) (Sadanandam et al., 2013; Hoadley et al., 2014; Roepman et al., 2014). The CRC Subtype Consortium (CRCSC) identified four robust consensus molecular subtypes (CMSs) using RNA-sequencing numbers from primary tumor samples derived from patients with early-stage colon cancer: CMS1, inflammation/immunity-rich genes; CMS2, normative; CMS3, metabolic; and CMS4, mesenchymal (Fontana et al., 2019). Seven DNA methylation subgroups were constructed based on DNA methylation in colon adenocarcinoma patients (Yang et al., 2019). However, there is disagreement among these classifications. Many attempts have been made to reach a consensus in classifying CRC subtypes, and such efforts play a critical part in determining the prognostic and predictive factors for colon cancer patients and in guiding treatment (Bramsen et al., 2017). Currently, due to the difficulty and cost of experimental verification, there is no general consensus on subclassification, and reliable molecular subtype methods are still needed to reveal the clinical potential of these subclassifications. This study developed an accurate method for determining molecular subtypes based on invasion-related genes.

Gao et al. built gene signature sets based on eight cancer hallmarks to predict the recurrence of stage 2 colon cancer in patients treated with fluorouracil-based chemotherapy (Gao et al., 2016). An “invasiveness” gene signature is associated with metastasis-free survival and OS in patients with medulloblastoma, lung cancer, or prostate cancer (Liu et al., 2007). However, “invasive” genetic markers in colon cancer have not been studied. In this work, we retrospectively identified four bone metastasis-related genes (INHBB, RBP7, RTN2, and ATOH1) and constructed a gene expression signature model for colon cancer patients by bioinformatic analysis. As a protein-coding gene, inhibin subunit beta B (INHBB) is involved in the synthesis of transforming growth factor-β (TGF-β) family members. INHBB expression has been found to be upregulated in colorectal cancer tissues and to be positively related to stromal and immune scores (Yuan et al., 2020). High RBP7 expression has been confirmed to be an independent biomarker for poor cancer-specific survival in patients with late- or early-stage colon cancer. Moreover, a study showed that ectopic expression of RBP7 could enhance the invasion and migration of colon cancer cells (Elmasry et al., 2019). In colon cancer tissues, positive expression of ATOH1 is closely related to a lower grade, a lower TNM stage, and better overall survival (Yang et al., 2018).

The spatial cellular expression patterns of RTN2 have not been investigated in colon cancer, but RTN2 expression was found to be positively correlated with degenerative disorder (Montenegro et al., 2012). Thus, we speculated that RTN2 could act as an anticancer target and a biomarker for the prognosis of colon cancer. The current findings indicate that the four-gene signature is an effective marker for predicting the survival prognosis of colon cancer patients.

There were still limitations to the current research, as we studied only the mRNA expression of genes, which is not always related to their specific biological activities. Second, the detailed mechanism still needs to be investigated in further experiments.

## Conclusion

In summary, we identified two new prognostic subtypes with significant differences in predicting colon cancer patient survival according to gene expression data from TCGA. Furthermore, we constructed a risk score model derived from four genes to predict the prognosis of colon cancer patients. This study suggests that gene expression profiles show the molecular characteristics of different subsets of colon cancer. Our results could facilitate the design of future clinical trials to identify colon cancer patients who could benefit from adjuvant chemotherapy.

## Data Availability Statement

The datasets presented in this study can be found in online repositories. The names of the repository/repositories and accession number(s) can be found in the article/Supplementary Material.

## Author Contributions

YD designed the study, reviewed, and edited the manuscript. TS and ZC contributed to the literature search. HJ contributed to the data acquisition. XZ contributed to the statistical analysis. TS wrote the initial draft of the manuscript. All authors read and approved the manuscript.

## Conflict of Interest

The authors declare that the research was conducted in the absence of any commercial or financial relationships that could be construed as a potential conflict of interest.

## Publisher’s Note

All claims expressed in this article are solely those of the authors and do not necessarily represent those of their affiliated organizations, or those of the publisher, the editors and the reviewers. Any product that may be evaluated in this article, or claim that may be made by its manufacturer, is not guaranteed or endorsed by the publisher.

## Footnotes

1
https://portal.gdc.cancer.gov/

2
https://www.ncbi.nlm.nih.gov/geo/

3
https://www.gsea-msigdb.org/gsea/index.jsp
