# Supplementary figures and images for: Identification of Distinct Molecular Patterns and a Four-Gene Signature in Colon Cancer Based on Invasion-Related Genes
Source: Front Genet. 2021 Aug 6;12:685371. doi: 10.3389/fgene.2021.685371 (PMC8378182; doi:10.3389/fgene.2021.685371)

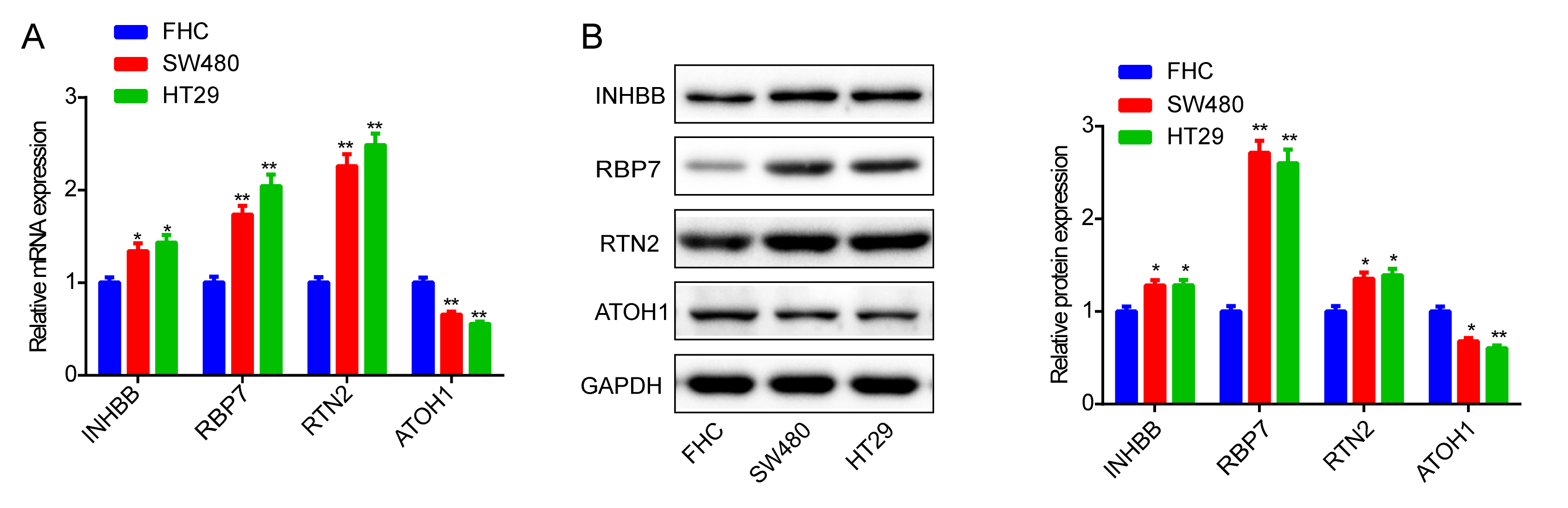

Supplement: Supplementary file 1 [file Image_1.tif]

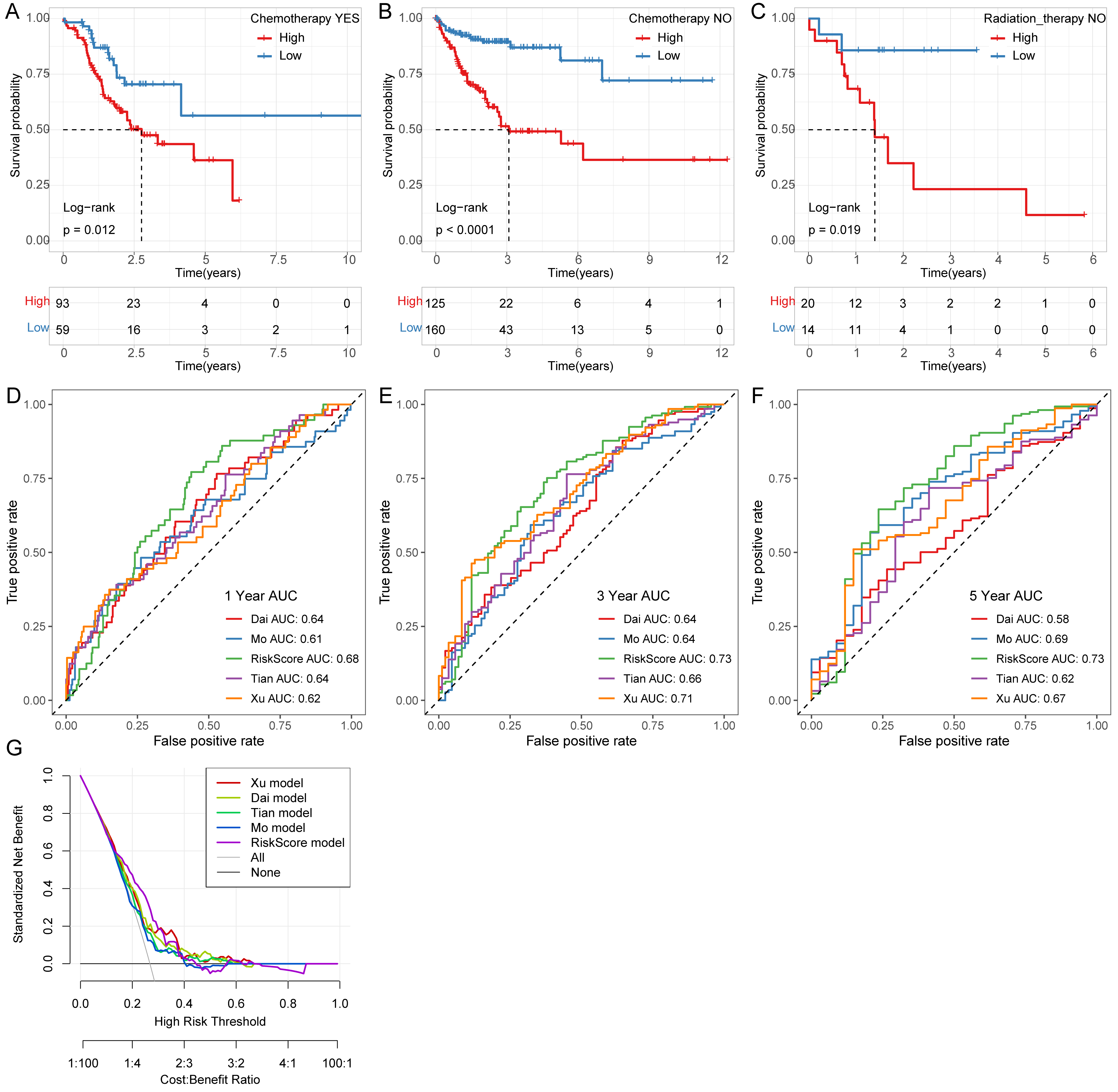

Supplement: Supplementary file 2 [file Image_2.tif]
