# Supplementary material for: Identification of Distinct Molecular Patterns and a Four-Gene Signature in Colon Cancer Based on Invasion-Related Genes
Source: Front Genet. 2021 Aug 6;12:685371. doi: 10.3389/fgene.2021.685371 (PMC8378182; doi:10.3389/fgene.2021.685371)
Supplement: Supplementary file 3 [file Table_1.docx]

**Table S1. Univariable and Multivariable analysis of Feature in GSE17538 dataset.**

| Feature | Univariable analysis | | | | Multivariable analysis | | | |
| --- | --- | --- | --- | --- | --- | --- | --- | --- |
|  | HR | 95% CI of HR | | *P* | HR | 95% CI of HR | | *P* |
|  |  | lower | upper |  |  | lower | upper |  |
| Age | 0.692 | 0.375 | 1.275 | 0.238 | 0.758 | 0.394 | 1.458 | 0.407 |
| Gender | 0.982 | 0.536 | 1.799 | 0.952 | 0.922 | 0.487 | 1.747 | 0.803 |
| Stage | 3.064 | 1.568 | 5.987 | 0.001 | 2.089 | 1.034 | 4.221 | 0.040 |
| Grade | 2.341 | 1.067 | 5.136 | 0.034 | 1.736 | 0.785 | 3.838 | 0.173 |
| RiskType | 3.386 | 1.701 | 6.741 | 0.001 | 3.117 | 1.463 | 6.643 | 0.003 |
