# Supplementary material for: Identification of Distinct Molecular Patterns and a Four-Gene Signature in Colon Cancer Based on Invasion-Related Genes
Source: Front Genet. 2021 Aug 6;12:685371. doi: 10.3389/fgene.2021.685371 (PMC8378182; doi:10.3389/fgene.2021.685371)
Supplement: Supplementary file 4 [file Table_2.docx]

**Table S2. Univariable and Multivariable analysis of Feature in GSE38832 dataset.**

| Feature | Univariable analysis | | | | Multivariable analysis | | | |
| --- | --- | --- | --- | --- | --- | --- | --- | --- |
|  | HR | 95% CI of HR | | *P* | HR | 95% CI of HR | | *P* |
|  |  | lower | upper |  |  | lower | upper |  |
| Stage | 3.939 | 2.780 | 5.860 | 0.054 | 2.430 | 1.121 | 3.875 | 0.180 |
| RiskType | 2.718 | 1.026 | 7.202 | 0.004 | 2.563 | 1.430 | 4.356 | 0.036 |
